# Supporting dataset and methods for egg sizes, eggshell thicknesses and metal concentrations measured in the shells and contents of eggs of Capercaillies *Tetrao urogallus*

**DOI:** 10.1016/j.dib.2019.103903

**Published:** 2019-04-06

**Authors:** Grzegorz Orłowski, Dorota Merta, Przemysław Pokorny, Ewa Łukaszewicz, Wojciech Dobicki, Janusz Kobielski, Artur Kowalczyk, Zenon Rzońca, Andrzej Krzywiński

**Affiliations:** a Institute for Agricultural and Forest Environment, Polish Academy of Sciences, Bukowska 19, 60-809 Poznań, Poland; b Department of Ecology and Environmental Protection Pedagogical University of Kraków, Podbrzezie 3, 31-054 Kraków, Poland; c Department of Hydrobiology and Aquaculture, Wrocław University of Environmental and Life Sciences, Chełmońskiego 38C, 51-630 Wrocław, Poland; d Division of Poultry Breeding, Wrocław University of Environmental and Life Sciences, Chełmońskiego 38C, 51-630 Wrocław, Poland; e Ruszów Forest Inspectorate, Leśna 2, 59-950 Ruszów, Poland; f Wisła Forest Inspectorate, Czarne 6, 43-460 Wisła, Poland; g Wildlife Park Kadzidłowo, Kadzidłowo 2, 12-220 Ruciane Nida, Poland

## Abstract

The dataset presented in this data paper supports “Eggshell resorption, and embryonic mobilization and accumulation of calcium and metals in eggs of wild and captive Capercaillies *Tetrao urogallus*” (Orłowski et al., 2019) [1]. Here we present the supplementary data on the following: (1) egg sizes, regional eggshell thicknesses (at the equator, sharp pole and blunt pole) as well as the concentrations of two major micronutrients (Ca and Mg) and eight trace elements (Cr, Cu, Mn, Fe, Co, Cd, Pb and Zn), measured in the shells and contents of eggs of captive-bred and wild Capercaillies. (2) How the proportions of elements sequestered into eggshells become depleted during embryogenesis expressed as the %change of concentrations of various elements measured in the shells and contents of eggs at different stages of embryonic advancement (early dead embryos, late dead embryos and hatched eggs). (3) The relationships between the age of dead embryos and three regional eggshell thicknesses and concentrations of different elements measured in the shells and contents of these eggs.

**Table:** Specifications table

Subject area	Ecology, Biological Sciences, Biogeochemistry, Developmental Biology
More specific subject area	Biogeochemistry of avian eggs; Eggshell traits; Eggshell elements.
Type of data	Tables
How data was acquired	Through field work and laboratory work
Data format	Raw, filtered and analysed
Experimental factors	Investigation of 38 eggs of wild Capercaillies and 133 eggs of captive-bred Capercaillies.
Experimental features	The ageing of embryos and measurement of egg sizes, regional eggshell thicknesses (at the equator, sharp pole and blunt pole) and concentrations of metals in the shells and contents of eggs.
Data source location	Eggs of wild Capercaillies derived from hens caught in the Arvidsjaur region of northern Sweden (Lapland: 65°35′31.5″N, 19°10′49.0″E), and from two lowland Polish populations (BoryDolnośląskie and PuszczaAugustowska). Eggs of captive-bred Capercaillies from three Polish breeding centres specializing in the rearing of the species (Wisła, Leżajsk and Kadzidłowo).
Data accessibility Related research article	The data are given in this article G. Orłowski, D. Merta, P. Pokorny, E. Łukaszewicz, W. Dobicki, J. Kobielski, A. Kowalczyk, Z. Rzońca, A. Krzywiński, Eggshell resorption, and embryonic mobilization and accumulation of calcium and metals in eggs of wild and captive Capercaillies *Tetrao urogallus*. Environ. Pollut., 249, 2019, 152-162.

**Table:** 

**Value of the data** • The data relate to egg breadth, three regional eggshell thicknesses and concentrations of 10 chemical elements measured in the shells and contents of eggs of captive-bred and wild Capercaillies. • Concentrations of two major micronutrients (Ca and Mg) and eight trace elements (Cr, Cu, Mn, Fe, Co, Cd, Pb and Zn) were measured in the shells and contents of eggs at different stages of embryonic advancement. • The data in this article provide information on the full scale of the magnitude of changes in concentrations of chemical elements in eggshells and egg contents between two extremes of avian embryonic development. • These data, clearly showing that metal levels in eggshells and egg contents vary in accordance with the state of embryonic advancement, are of critical importance for in-depth assessments of the sources of variation of egg/eggshell quality in environmental studies of avian eggs.

## 1 Data

The data presented here constitute the basis for the article by Orłowski et al. [1]. The dataset provides detailed information on: (1) egg sizes, regional eggshell thicknesses and the levels of two major micronutrients (Ca and Mg) and eight trace elements (Cr, Cu, Mn, Fe, Co, Cd, Pb and Zn), measured in the shells and contents of eggs of captive-bred and wild Capercaillies (Table 1, Table 2). (2) How the proportions of elements sequestered into eggshells become depleted during embryogenesis expressed as the %change of concentrations of various elements measured in the shells and contents of eggs at different stages of embryonic advancement (early dead embryos, late dead embryos and hatched eggs) (Table 3). (3) The relationships between the age of dead embryos and three regional eggshell thicknesses and concentrations of different elements measured in the shells and contents of these eggs (Table 4) (see Table 4).

**Table 1:** Summary descriptive statistics of egg breadth, three regional eggshell thicknesses (measured with the inner shell membrane) at the equator, sharp pole and blunt pole, and concentrations of different elements measured in the shells and contents of eggs of captive-bred Capercaillies *Tetrao urogallus* (*n* = 133 eggs) and wild Capercaillies (*n* = 38 eggs).

Egg characteristic/element concentration (unit)	Captive-bred Capercaillies*						Wild Capercaillies					
	*n*	Mean	−95% CI	+95% CI	Min	Max	*n*	Mean	−95% CI	+95% CI	Min	Max
Egg breadth (mm)	102	41.4	41.1	41.6	36.4	44.1	19	40.6	40.0	41.1	36.6	42.1
Equatorial eggshell thickness (mm)^a^	151	0.42	0.40	0.42	0.27	0.55	38	0.31	0.30	0.32	0.25	0.37
Sharp pole eggshell thickness (mm)	116	0.36	0.35	0.37	0.24	0.50	26	0.31	0.29	0.33	0.22	0.41
Blunt pole eggshell thickness (mm)	117	0.33	0.32	0.34	0.15	0.52	25	0.29	0.28	0.31	0.22	0.38
Eggshell Cr (ppm d.w.)	133	0.27	0.21	0.32	0.001	1.44	38	0.17	0.11	0.22	0.001	0.65
Eggshell Cu (ppm d.w.)	133	0.61	0.53	0.69	0.001	3.12	38	0.42	0.35	0.50	0.16	1.16
Eggshell Mn (ppm d.w.)	133	9.30	8.37	10.24	0.32	29.13	38	5.92	5.15	6.69	1.61	10.98
Eggshell Fe (ppm d.w.)	133	51.38	45.80	56.95	16.52	200.7	38	27.78	23.57	32.00	18.30	82.18
Eggshell Co (ppm d.w.)	132	14.13	13.83	14.42	8.63	17.09	38	12.38	11.80	12.96	8.65	15.92
Eggshell Cd (ppm d.w.)	133	0.09	0.09	0.10	0.001	0.21	38	0.09	0.06	0.11	0.001	0.32
Eggshell Pb (ppm d.w.)	30	0.37	0.36	0.39	0.32	0.47	23	0.38	0.36	0.41	0.32	0.56
Eggshell Zn (ppm d.w.)	133	7.73	6.68	8.77	0.72	31.34	38	3.64	2.10	5.19	0.23	29.10
Eggshell Mg (ppm d.w.)	132	7832	7402	8263	3556	19839	38	13496	9471	17521	5000	84004
Eggshell Ca (ppm d.w.)	132	313052	299463	326642	201978	582599	38	392921	367331	418511	186995	559188
Egg contents Cr (ppm d.w.)	103	0.76	0.71	0.82	0.17	2.24	19	0.66	0.57	0.76	0.31	1.04
Egg contents Cu (ppm d.w.)	103	3.29	3.14	3.43	1.13	5.24	19	4.01	2.98	5.04	2.78	11.02
Egg contents Mn (ppm d.w.)	103	3.84	3.51	4.18	1.27	10.73	19	3.45	2.70	4.19	0.80	5.67
Egg contents Fe (ppm d.w.)	103	130.2	124.2	136.2	57.6	237.2	19	124.6	111.0	138.3	71.2	155.6
Egg contents Co (ppm d.w.)	103	1.75	1.69	1.80	1.07	2.58	19	1.72	1.64	1.80	1.46	1.95
Egg contents Cd (ppm d.w.)	103	0.81	0.79	0.83	0.41	1.13	19	0.89	0.87	0.91	0.83	0.95
Egg contents Pb (ppm d.w.)	103	0.28	0.27	0.29	0.18	0.33	19	0.31	0.31	0.32	0.29	0.34
Egg contents Zn (ppm d.w.)	103	87.6	83.3	91.9	38.3	182.4	19	87.6	76.8	98.3	63.0	138.9
Egg contents Mg (ppm d.w.)	103	435	412	458	123	934	19	440	390	491	306	703
Egg contents Ca (ppm d.w.)	103	3538	2941	4135	1122	20432	19	3141	2392	3890	1910	7054

**Additional comment of data in**Table 1**.** Despite the larger size of wild Capercaillie hens (Table 1), the difference in resource levels in the diet/environment [25], [26] or nutrient limitation [27] seem to be the major driver explaining the smaller size of these eggs and the resulting thinner shells. In the Capercaillie (similar to the overall trend reported across other birds [28]), eggshell thickness is a function of egg size, and larger eggs tend to have thicker shells [13], especially as captive-bred Capercaillie hens were offered a supplementary diet, which generally increases the size of eggs laid by precocial females [29], [30]. The difference in egg size between wild (high northern latitudes) and captive-bred Capercaillies could also have been due, e.g. to variations related to laying order or clutch size [29], [31], [32] or temperature/weather conditions at a breeding site [27].

*Note: The wild Capercaillie hens (*n* = 14) were 18% heavier (weighed in Sweden immediately after trapping) than the hens bred in captivity (*n* = 15; weighed at different times of the breeding season, April–July): on average 2166 g (range = 1867–2383 g) vs 1828 g (1547–1988 g), respectively (Mann-Whitney test, Z = 4.2, *P* < 0.001) (D. Merta – unpubl.).

a To increase the sample size we used additional measurements of equator thickness made on 20 post-hatched eggshells from the centre in Wisła.

**Table 2:** Concentrations of 10 chemical elements measured in post-hatched eggshells of wild Capercaillies *Tetrao urogallus* from northern Sweden (*n* = 9 eggshells) and from two breeding areas in Poland (Puszcza Augustowska [*n* = 7] and Bory Dolnośląskie [*n* = 3]), and also of captive breed Capercaillies from two Polish breeding centres (Leżajsk [*n* = 14] and Kadzidłowo [*n* = 16]); the various superscripts denote significant differences in the *post-hoc* comparison between the five locations.

Element	Wild Capercaillies			Captive-bred Capercaillies		Kruskal-Wallis test on difference	
	Northern Sweden	BoryDolnośląskie (SW Poland)	PuszczaAugustowska (NE Poland)	Leżajsk	Kadzidłowo	H (df = 4 and 49)	*P*-value
Cr	0.16 (±0.05)	0.15 (±0.11)	0.30 (±0.10)	0.09 (±0.03)	0.21 (±0.07)	4.51	0.342
Cu	0.38 (±0.02)^A^	0.24 (±0.04)	0.34 (±0.02)	0.30 (±0.01)^B^	0.34 (±0.02)	12.37	0.015
Mn	3.73 (±0.41)^A^	6.45 (±0.58)	7.04 (±0.80)^AB^	2.73 (±0.37) ^AC^	7.70 (±0.80)^B^	30.67	<0.0001
Fe	24.45 (±1.68)	20.37 (±0.93)	22.88 (±1.46)	29.15 (±2.97)	37.33 (±4.34)	14.12	0.007
Co	10.53 (±0.39)	10.43 (±0.77)	12.36 (±0.62)	11.13 (±0.27)	11.86 (±0.40)	8.30	0.081
Cd	0.084 (±0.002)^A^	0.091 (±0.005)^AB^	0.072 (±0.002)^CD^	0.067 (±0.002)^C^	0.077 (±0.001)^AD^	30.04	<0.0001
Pb	0.35 (±0.01)	0.36 (±0.01)	0.36 (±0.01)	0.39 (±0.01)	0.35 (±0.01)	8.16	0.086
Zn	3.26 (±0.50)	2.64 (±0.57)	1.97 (±0.32)	2.37 (±0.31)	2.13 (±0.27)	7.42	0.115
Mg	12580 (±643)^A^	35429 (±24308)	10575 (±212)	9822 (±280)^B^	11278 (±484)	13.65	0.009
Ca	391943 (±6392)^A^	422510 (±3892) ^AB^	329319 (±6326)^C^	331534 (±3281)^C^	369420 (±5543) ^AD^	36.10	<0.0001

**Table 3:** %Change of concentrations of various elements measured in the shells and contents of eggs at different stages of embryonic advancement in captive-bred and wild Capercaillies *Tetrao urogallus*; early dead embryos (0–7 days), late dead embryos (18–26 days) and hatched eggs (27 day = post-hatch eggshells). For element concentrations, see Fig. 3 in Ref. [1] and Table 1.

Element	Captive-bred Capercaillies		Wild Capercaillies
	Rotten eggs vs hatched	Early dead vs hatched	Early dead vs hatched
Eggshell Cr	−53	**−**43	+92
Eggshell Cu	−62	**−**50	−34
Eggshell Mn	−55	**−**44	−15
Eggshell Fe	−46	**−**35	−28
Eggshell Co	−24	**−**23	−18
Eggshell Cd	−43	**−**20	−16
Eggshell Pb	n.d.	n.d.	−30
Eggshell Zn	−80	**−**70	−44
Eggshell Mg	+35	+53	+35
Eggshell Ca	+2	+19	−7
	Rotten eggs vs late dead	Early dead vs late dead	
Egg contents Cr	+85	+71	n.d.
Egg contents Cu	+37	+29	n.d.
Egg contents Mn	+63	+63	n.d.
Egg contents Fe	+54	+46	n.d.
Egg contents Co	+34	+32	n.d.
Egg contents Cd	+23	+18	n.d.
Egg contents Pb	+15	+11	n.d.
Egg contents Zn	+32	+26	n.d.
Egg contents Mg	+59	+47	n.d.
Egg contents Ca	+285	+248	n.d.

**Table 4:** Spearman correlation coefficients (*r*_s_) testing the relationship between the age of dead embryos (in days) and three regional eggshell thicknesses (at the equator, sharp pole and blunt pole) and concentrations of different elements (ppm d.w.) measured in the shells and contents of eggs (see Fig. 2 in Ref. [1]) of captive-bred and wild Capercaillies *Tetrao urogallus*. The analysis used data of infertile eggs, rotten eggs (in most cases infertile; both aged as day 0) and hatched eggs (= post-hatch eggshells) aged as day 27. The “all eggs” category comprises data from 38 eggs of wild Capercaillies, i.e. 16 infertile eggs, three eggs with embryos dead at days 5, 7 and 15, and 19 hatched eggs. Note: Due to low sample size and unequal distribution of developmental stages results for eggs of wild Capercaillies should be treated with caution.

	Eggs of captive-bred Capercaillies			Eggs of wild Capercaillies			All eggs		
	*r*_s_	*P*-value	*n*	*r*_s_	*P*-value	*n*	*r*_s_	*P*-value	*n*
Equatorial eggshell thickness	−0.604	<0.001	124	−0.173	0.299	38	−0.491	<0.001	162
Sharp pole eggshell thickness	−0.246	0.010	109	−0.359	0.072	26	−0.206	0.016	135
Blunt pole eggshell thickness	−0.022	0.816	110	0.512	0.009	25	−0.103	0.235	135
Eggshell Cr	−0.167	0.062	126	0.170	0.308	38	−0.101	0.200	164
Eggshell Cu	−0.370	<0.001	126	−0.196	0.230	38	−0.364	<0.001	164
Eggshell Mn	−0.292	0.001	126	−0.169	0.266	38	−0.284	<0.001	164
Eggshell Fe	−0.302	0.001	126	−0.222	0.180	38	−0.316	<0.001	164
Eggshell Co	−0.552	<0.001	126	−0.662	0.000	38	−0.564	<0.001	164
Eggshell Cd	−0.301	0.001	126	0.131	0.546	38	−0.211	0.007	164
Eggshell Zn	−0.428	<0.001	126	−0.073	0.665	38	−0.395	<0.001	164
Eggshell Mg	0.398	<0.001	126	0.184	0.270	38	0.378	<0.001	164
Eggshell Ca	0.154	0.084	126	−0.211	0.203	38	0.119	0.131	164
Egg contents Cr	0.391	<0.001	96	0.077	0.753	19	0.369	<0.001	115
Egg contents Cu	0.171	0.096	96	−0.019	0.937	19	0.082	0.385	115
Egg contents Mn	0.353	<0.001	96	−0.359	0.131	19	0.282	0.002	115
Egg contents Fe	0.289	0.004	96	−0.225	0.354	19	0.236	0.011	115
Egg contents Co	0.266	0.009	96	0.195	0.424	19	0.237	0.011	115
Egg contents Cd	0.185	0.070	96	0.495	0.031	19	0.081	0.392	115
Egg contents Pb	0.259	0.011	96	0.231	0.342	19	0.069	0.465	115
Egg contents Zn	0.266	0.009	96	0.032	0.897	19	0.227	0.015	115
Egg contents Mg	0.099	0.337	96	0.191	0.435	19	0.089	0.347	115
Egg contents Ca	0.324	0.001	96	−0.059	0.809	19	0.254	0.006	115

## 2 Experimental design, materials and method

### 2.1 Study species, and origin of eggs to be analysed

The Capercaillie is the largest grouse species in the world (body weight of females – 1400–2500 g; males – 3300–4800 g). It is a sedentary species, inhabiting the boreal and temperate forest zones of Eurasia, predominantly coniferous and continental, with a strong preference for areas with extensive, well-grown tree stands [2]. During the last few decades, populations of Capercaillie have declined throughout its range [3]. Although the species is not considered to be globally threatened, many local populations in central and western Europe (like those in Poland) have become extinct (reviewed in Ref. [4]), while remaining small, isolated populations are threatened [5]. Capercaillies still occur in considerable numbers only in the boreal forests of Fennoscandia and Russia, although population declines have also been observed in these regions [3], [4], [6]. For restocking or re-establishing local populations of Capercaillies, the release of birds reared in captivity or caught in the wild has become a common conservation tool in many European countries, including Poland [7], [8].

Fourteen wild Capercaillie hens were caught in the northern Sweden were transported to Poland by air in order to minimize stress (flight time approximately 4 hours). In order to acclimate them to local conditions in Poland for the required quarantine period, the hens were placed in roughly circular adaptation aviaries (area 20 m^2^; height 1 m). These were placed in a suitable habitat, dominated by Scots pine *Pinus sylvestris* stands with well-developed ground vegetation dominated by bilberry *Vaccinium myrtillus*, far away from built-up/populated areas [9]. The translocated hens were very shy and flew into the net around the aviary on being frightened by approaching people, so a daily search for their eggs was not possible. During their first 2.5–3 weeks in the quarantine aviaries, these hens laid 48 eggs; they consumed mostly natural food picked from the ground, i.e. the local vegetation. Some eggs (*n* = 12) were laid singly, in random places; these were subsequently incubated by Domestic Chickens or artificially in incubator, but ultimately failed to hatch. Seven hens set up nests and three of them produced offspring (*n* = 9). The translocated hens were not stimulated to lay more eggs by having their eggs removed from the nests; only abandoned eggs were taken away.

As reported in females of other grouse species [10], Capercaillie hens can store Ca for a relatively long time (i.e. weeks or months) prior to reproduction (through the formation and subsequent utilization of medullary bone) and then mobilize it during egg laying. During the egg-laying period in Poland, the Swedish birds may have depleted the resources they had derived from their original breeding sites in Scandinavia. Presumably, therefore, the contents of Ca and other elements in the eggs laid in the acclimation aviaries were not affected by their diet in captivity. Moreover, contact between the wild-caught hens with males (copulation) took place only in Sweden; Capercaillie hens remain fertile for up to 29 days after separation from males [11].

The eggs and post-hatched eggshells of captive-bred Capercaillies were obtained from three Polish breeding centres specializing in the rearing of the species: Wisła (49°32′05.4″N, 18°55′58.1″E), Leżajsk (50°15′23.6″N, 22°18′20.2″E) and Kadzidłowo (52°13′46.8″N, 21°0′44.0″E). These breeding centres are situated a long distance from human settlements. The breeding flocks of Capercaillies at these centres consisted of 2–11 males and 15–35 (53 in total) females. The birds were kept in aviaries, and in spring and summer were able to roam around a large fenced yard with trees, bushes and natural ground flora including bilberry [9], [12]. At all the breeding centres, the birds were fed a variety of natural food (fruits, leaves and cereal seeds) and had unrestricted access to a variety of natural vegetation and invertebrates. During the reproductive period, the hens' diet was supplemented with pigeon grit (for more details, see Refs. [12], [13], [14]). The bird keepers monitored the nests and eggs, and sometimes removed a few eggs during the initial laying period, inducing the hens to lay more. Once egg incubation began, the females were no longer disturbed by the keepers [12]. The first-laid eggs were usually pre-incubated by domestic hens for 21–22 days before being placed in the hatcher, or else they were incubated only in the incubators [15].

Our analysis included fragments of eggshells from wild-nesting Capercaillies (from *c.* six hens in total): nine post-hatched shells from the eggs of females caught in Sweden (after successful breeding attempts in captivity) and three post-hatched shells from two hens (equipped with radio transmitters) after one year of free living in the Bory Dolnośląskie (51°22′N, 15°6′E). We also sampled seven post-hatched shells (one brood) from wild Capercaillies living in the Augustowska Primeval Forest (53°50′N, 23°6′E), north-eastern Poland. Unhatched eggs (*n* = 21) of wild-nesting Capercaillies derived from hens shortly after their translocation from Sweden to Poland.

The eggs of captive-bred birds (laid in spring 2014) included intact unhatched eggs (*n* = 103) and post-hatched eggshells (*n* = 50), which preceded incubation by Capercaillie females, domestic hens or in artificial incubators. These eggs included 103 intact ones from females kept at the Wisła breeding centre (the majority of eggs were obtained from 39 different hens). Post-hatched eggshells (*n* = 50) of captive-bred birds include: 16 post-hatched shells from Kadzidłowo (including 6 shells from one Swedish hen reared for one year in captivity; all the eggs obtained from 10 different hens) and 14 post-hatched shells from Leżajsk (from 4 different hens), and 20 eggshells from Wisła (for which only the measurements of equator thickness were made; see Table 1). Most eggs from Kadzidłowo and Leżajskhad been laid by Capercaillie hens originally from Belarus. The eggs were collected over the entire laying season, so that no one part of the season was favoured over any other; they thus represent different positions in the laying sequences of the various hens.

### 2.2 Egg processing and ageing of embryos

The maximum width (in mm) of most intact eggs of captive-bred birds were measured using an electronic calliper. The unhatched eggs were opened to determine their status, to age the dead embryos and to measure the eggshell thickness. The entire contents of the eggs were emptied into Eppendorf tubes and frozen (at −20 °C) until further processing. After drying, the eggshells were stored in plastic containers at room temperature. The eggshell thickness was measured with the attached inner shell membrane to the nearest 0.001 mm using a micrometer (IP65 125–150 mm HOGETEX) in three regions of the egg: the equator (the widest part of the egg; four measurements were made, then averaged to obtain the overall equatorial eggshell thickness); the blunt pole and the sharp pole (a single measurement each). As we defined the poles as the exact central peaks at the ends of the egg, our single measurements at these points presumably do not include potential variations in shell thickness over broader areas of both egg ends that might result from irregularities in the internal shell surface or minerals eroded from the mammillary cones due to embryonic depletion, or even the presence of a pigment spot. It should acknowledged, that previously, the blunt pole and the sharp pole of an egg (and its eggshell thickness measurement) could have referred to as much as *c.* 1/5 of the entire egg length (cf [16]). To the best of our knowledge, however, there are no prior formal recommendations or studies stipulating where the boundaries between the various regions of an egg along its longitudinal section should be delineated.

Fragments of post-hatched eggshells were assigned to one of these three egg regions on the basis of the spherical shape of intact eggs.

The age at death of the embryo in each individual egg was assigned by two co-authors (see the main article). A recent investigation analysed the rate of infertility and developmental status of another sample of Capercaillie eggs from the Wisła breeding centre (sampled in 2015). There, besides the similar criteria for ageing dead embryos, artificially coloured blastodermal cells were identified (*sensu* [17]) and 146 (74%) of 196 unhatched eggs were infertile [13]. We also used rotten eggs in our analysis, but the embryos in these could not be aged owing to the putrefaction of their contents. The majority of rotten eggs were presumably infertile or the embryos in them had died at an early stage (<3 days), as visual inspection of their contents revealed no sign of an embryo; putrefaction in infertile/non-embryonated eggs had most likely started early, and continued during the artificial incubation. As all the rotten eggs were from captive-bred Capercaillies from Wisła breeding centre, we placed special emphasis on comparing these eggs with those in later stages of embryonic development (see Results in Ref. [1]); this was mostly because of potential post-mortem changes in the shell and/or egg contents between the time when the embryos died and when the eggs were later opened [[18], [19]; see also Introduction in Ref (1)]. Overall, however, we expected that the rotten eggs, because of the absence of developing embryos and the resulting depletion of the inner eggshell layer, would have thicker shells [20], [21] and higher concentrations of Ca and other micronutrients than eggs with more advanced embryos or post-hatched eggshells. For the particular requirements of this work, we allocated the eggs to five age classes: rotten eggs, and four classes of eggs in successive stages of embryonic development: early dead eggs (0–7 days) comprised eggs without any visible sign of an embryo (most probably infertile eggs) and eggs with early dead embryos aged ≤7 days; eggs with midterm dead embryos (8–17 days); eggs with late dead embryos (18–26 days); and hatched eggs (27 days = post-hatch eggshells). The rationale for pooling “early dead eggs”, eggs without visible embryos (*n* = 12) and eggs with dead embryos ≤7 days (*n* = 24) into one category was that none of the traits analysed (eggshell thicknesses and eggshell/egg content metal levels in the eggs of captive-bred Capercaillies) differed between these two egg samples (ANOVA, *P* ≥ 0.063). In captivity, Capercaillie hens incubated their eggs for 25–28 days [12].

### 2.3 Chemical analysis of egg shells and contents

After having thawed out (October–November 2016), the entire contents of an egg, i.e. yolk, albumen and embryos and *c.* one-third of an entire eggshell were examined at the Department of Hydrobiology and Aquaculture of the Wrocław University of Environmental and Life Sciences (by two co-authors, PP, WD). The eggshell samples were analysed with the shell membrane attached. Prior to chemical analysis, all the eggshells were washed twice with water containing detergent and rinsed with twice-distilled water. Embryos were present only in eggs <20 days old. For chemical analysis we cut out from intact eggs an oval piece containing the equatorial part of an egg (without the shell regions from the blunt/sharp poles). In the case of post-hatched eggshells (mostly from nests of wild Capercaillies, when only very small eggshell fragments were present) we took all the available fragments to obtain a sample mass enabling chemical analysis. Any discrepancies between sampling shells from various egg regions and the potential influence of this on the results of the chemical analysis are commented on in the Discussion in Ref. [1].

### 2.4 Statistical analyses

The statistical analyses were performed using Statistica ver. 7.0 [22] and Excel software. The alpha threshold was 0.05.

We used data representing two eggs groups of different origin: 38 eggs of wild Capercaillies and 133 eggs of captive-bred birds. Sample sizes varied for individual egg-size related traits and metal concentrations. The eggs classified as ‘wild’ were obtained from *c.* 14–19 different wild Capercaillie hens; and eggs of captive-bred Capercaillies obtained from *c.* 53 hens (see above). Because two or more hens were reared communally in the aviaries, precise assignment of both categories of eggs (i.e. wild and captive) to an individual female was impossible. For instance, only one egg could be assigned to a specific individual female translocated from Sweden. This factor could not therefore be controlled for in the analysis. Moreover, both the variation in eggshell thicknesses and the concentrations of all 10 metals within the sample of seven post-hatched shells (one brood) from wild Capercaillies from the Augustowska Primeval Forest (see Table 2) were distributed normally (tested in Kolmogorov-Smirnov tests, in all cases *P* > 0.20). Therefore, to increase the sample size and to take into account this variability, using all the data points of this sample in our analysis might be justified from the point of view of data independence, and mitigate the issue of potential pseudoreplication [23].

Tukey's test with the Spjøtvoll-Stoline modification for unequal sample sizes was applied to obtain the *post-hoc* contrast for assessing the differences between the two egg classes (within ANOVA) [24]. To meet the assumption of normality (assessed by the Kolmogorov-Smirnov test), we log-transformed some of the element concentrations measured in egg shells and contents before the analysis. Egg classes with a single measurement were excluded from the ANOVA visualized in Ref. [1] in Figs. 1 and 3.

Spearman's rank correlation coefficients (*r*_s_) were used to test the relationship between the age of dead embryos (in days), and three regional eggshell thicknesses and concentrations of different elements measured in the shells and contents of both groups of eggs. The analysis included data relating to rotten eggs, which in most cases were probably infertile (aged as 0 days); and hatched eggs (= post-hatch eggshells) aged 27 days (see Fig. 2 in Ref. [1]).
